# Application of biological and single-strand conformation polymorphism assays for characterizing potential mild isolates of *Citrus tristeza virus* for cross protection

**DOI:** 10.1186/s13568-019-0903-5

**Published:** 2019-10-31

**Authors:** Sagheer Atta, Ummad ud din Umar, Muhammad Amjad Bashir, Abdul Hannan, Ateeq ur Rehman, Syed Atif Hasan Naqvi, Changyong Zhou

**Affiliations:** 1 National Citrus Engineering Research Center, Citrus Research Institute, Southwest University, Chongqing, 400712 China; 2 Department of Plant Protection, Faculty of Agricultural Sciences, Ghazi University, Dera Ghazi Khan, 32200 Pakistan; 3 Department of Plant Pathology, Faculty of Agricultural Sciences and Technology, Bahauddin Zakariya University, Multan, 60800 Pakistan; 4 Department of Botany, Ghazi University, Dera Ghazi Khan, 32200 Pakistan

**Keywords:** CTV, Indexing, DTBIA, SSCP, Mild strain cross protection

## Abstract

*Citrus tristeza virus* (CTV) by killing millions of citrus cultivars grown on sour orange rootstock worldwide has become one of the most dangerous viral pathogen. Characterization of 12 CTV isolates was analyzed by biological indexing. Infected samples of citrus were collected from citrus growing areas of Pakistan and CTV was detected by symptoms on indicator plants and confirmed by direct tissue blot immunoassay (DTBIA). CTV positive samples were graft inoculated on six biological indicator hosts in the study. A standardized protocol was deployed to study biological characteristics of these isolates. All biological indicators induced mild and from mild to moderate reactions against all of the CTV isolates tested. About two isolates produced stem-pitting symptoms from moderate to severe on Mexican lime. CTV strains were further characterized and confirmed by the analysis of p25 gene of CTV isolates using single-strand conformation polymorphism (SSCP) assay. SSCP analysis revealed that most isolates confined only one predominant sequence variant. SSCP profiles of PCR amplified products from CTV isolates showed bands patterns corresponding to mild and sever strain. Three isolates (4MF, 8KBS and 10GS) from different regions and cultivars were identified as potential source of mild strains for cross protection. These results are the best base for mild strain cross protection (MSCP) in the country.

## Introduction

Pakistan is always considered among the top citrus producers of the world, and its production is consumed locally and mainly exported to fresh fruit markets. Hence, achieving high yields and best fruit quality are main steps to keep the citrus industry of Pakistan alive (Memon 2017). However, the presence of viruses and viroid diseases in the country are the limitations to production and quality of the produce (Atta et al. 2017; Wang et al. 2018). *Citrus tristeza virus* (CTV), a member of family Closteroviridae genus *clotserovirus*, is causal agent of economically most important disease of citrus which has caused death of about 100 million citrus trees around the globe (Moreno et al. 2008) and damage is increasing (Ferretti et al. 2014). It causes different disease outbreaks in the form of stem-pitting, quick decline, and stunting of tress are important one (Bar-Joseph et al. 1989). Different isolates or biological strains are involved which lead to variety of symptoms in infected plants. Standardized indicators are being employed to get biological properties of CTV isolates (Garnsey et al. 1991). Symptoms produced by the different CTV strains range from invisible to visible severe symptoms which include pits in the stems, branches and trunks, stunted growth, quick decline, seedling yellows, vein clearing and death of whole tree. Stem-pitting symptoms being the most conspicuous also have been identified in other plants like cherry and grape (Mircetich et al. 1977; Stouffer et al. 1969; Uyemoto et al. 1995), plum (Mircetich and Fogle 1969), peach (Barrat et al. 1968) (Garnsey et al. 1980; Mircetich and Fogle 1969; Mircetich et al. 1977) and apple (Hilborn et al. 1965; Tukey and Mink 1961). These stem-pitting diseases have been caused by or associated with viruses. A number of commercial cultivars such as sweet orange (*Citrus sinensis* L. Osbeck), grapefruit (*C*. *paradisi* Macfad), Kinnow’ and Feutrell’s Early’ mandarins (*C. reticulata* Blanco), lime (*C*. *aurantifolia* L. Swingle), pummelo (*C*. *grandis* L. Osbeck), and some pummelo hybrids induced stem-pitting symptoms when infected with CTV isolates (Atta et al. 2017). However, there is little confusion about the nature of stem-pitting induced by CTV strains. It is imperative that stem pitting symptoms induced by a CTV strain in one citrus cultivar may not induce in another citrus host. When tree is infected with CTV isolate that induce stem-pitting, it results in loss of vigor, size of fruit become small, and ultimately it declined. Stem-pitting is formed in the form of long narrow trenches in the xylem wood. It may also cause a rope-like appearance to tree trunk and twigs in severe form.

Grapefruit even grafted on tolerant rootstocks, when infected by CTV gave altered growth and development (Müller et al. 1996). Stunted growth, reduced development, small fruits and stem pitting in new branches and twigs are the most conspicuous symptoms produces by the tristeza diseases on different cultivars. Citrus diseases of CTV have been reported from all over the world and are endemic in Pakistan. Orange stem-pitting (OSP) isolates have been associated with the tree decline of sweet orange (Broadbent et al. 1992; Owen-Turner 1990). In Pakistan almost 50% sweet orange trees are affected by stem-pitting (Atta et al. 2011, 2012). Set of standardized citrus indicators grown under controlled conditions are being efficiently employed for Biological characterization of CTV strains (Garnsey et al. 1987b). It is substantial to analyze or characterize CTV isolates on molecular basis to acquire more information about the epidemic strains and to screen out mild strains (Ochoa et al. 2000; Yang et al. 1999). Molecular methods are also used to distinguish CTV isolates, e.g. RFLP analysis (Gillings et al. 1993), Single Strand Conformation Polymorphism (SSCP) analysis (RUBIO et al. 1996) and sequencing of selected genes (Pappu et al. 1993). SSCP analysis is an accurate and rapid technique to identify CTV stains and its biological characteristics (Yan et al. 2007). It accelerates the screening of mild strains of CTV. When selected isolates of mild CTV strain were inoculated the trees in field, it prolonged the productive life of citrus orchards and production of new plants on CTV-tolerant rootstocks. Nowadays, information is available to implement mild strain crop protection (MSCP) against CTV severe strains (Folimonova 2013). However, to get the benefit from our citrus industry we need to make our citrus groves productive and vigorous. In this regard, effort to do biological indexing for characterization of CTV for the identification mild strains which could help to develop mother plants with commercial potential. In this study, for good understanding on CTV population and the management of disease in Pakistan on the basis of biological characteristics of CTV isolates, biological indexing was approached for 12 CTV isolates. The sequence of isolate 21c was used in our previous study (Atta et al. 2017).

## Materials and methods

### Virus isolates

A survey was conducted for the collection of samples from symptomatic plants of different cultivars of citrus from Sargodha, Bhalwal, Sahiwal and Faisalabad areas of Punjab province of Pakistan (Table 1). These samples were tested with direct tissue blot immunoassay (DTBIA) (Garnsey et al. 1993). For further studies samples infected with CTV were maintained on Fenghuang pummelo and sweet orange. A standardized protocol was used for the evaluation of biological characteristics of the isolates (Garnsey et al. 1991).

**Table 1:** Biological indexing of CTV confirmed through DTBIA

CTV isolate	Cultivars	District of origin	Conformation by DTBIA
1AF	Acid lime	Faisalabad	ª+
2KF	Kinnow	Faisalabad	+
3MF	Mosambi	Faisalabad	+
21c	Mosambi	Faisalabad	+
4MF	Mosambi	Faisalabad	+
5MF	Mosambi	Faisalabad	+
6MF	Mosambi	Faisalabad	+
7KBS	Kinnow	Bhalwal/Sargodha	+
8KBS	Kinnow	Bhalwal/Sargodha	+
9KBS	Kinnow	Bhalwal/Sargodha	+
10GS	Grapefruit	Sargodha	+
11GS	Grapefruit	Sargodha	+

ª+: found positive by DTBIA

### Evaluation of tristeza symptomatology

Bark of the plants was peeled of from 15 cm long segments for the evaluation of stem-pitting symptoms. Each plant was sampled from all quadrants in four segments by making cut on young twigs. The presence of the stem-pitting, vein clearing, and seedling yellows symptoms were quantified by rating scale (Müller et al. 1996).

### Biological indexing

Six biological indicators were prepared.

1. Fenghuang pummelo on pummelo
2. Duncan grapefruit on sour orange.
3. Sour orange seedlings.
4. Mexican lime on tangerine and Fairchild.
5. Symons sweet orange on sour orange.
6. Symons sweet orange seedlings.

Mexican lime (*Citrus aurantifolia*) grafted on Fairchild mandarin (*Citrus reticulata* Blanco) and Tangerine (*Citrus *×* tangerina*) was used for symptoms expression of stem-pitting, vein clearing, and seedling yellows. Duncan grapefruit (*Citrus paradise* Macf. *cv.* Duncan) when grafted on Sour orange (*Citrus aurantium*) and sour orange seedlings gives best evaluation of seedling yellows (SY). Symons sweet orange (*Citrus sinensis* L. Osbeck cv. Symons) seedlings and Duncan grapefruit give best symptoms of stem-pitting. Sweet orange was grafted on sour orange (S/S) for the evaluation of stem-pitting, chlorosis, and stunting. While stem-pitting, chlorosis and leaf cupping were evaluated on Fenghuang pummelo (*Citrus grandis*) as these indicators depict best CTV symptoms (Broadbent et al. 1996; Garnsey et al. 1987b). Virus free seedlings were grown in 15 cm pots of UC mixture (50 peat: 50 sand with added fertilizer (Baker and Chandler 1957).

Five plants from each indicator host were graft inoculated with infected barks from each CTV isolate and five plants of each indicator host were left as un-inoculated control whereas five plants were grafted with healthy control. All grafted plants were kept in green house in a controlled day and night temperatures. 12 months after the graft inoculation, symptoms of stem pitting were observed on main stem and branches by removing bark, while foliar symptoms were checked visually as the new flushes and branches started emerging as: mild (+) (slight appearance of vein clearing); severe (++) (vein clearing); and very severe (+++) vigorous vein clearing till the point of vein impregnate and leaf curling). The rating of three plants was considered as average rating of the test.

### Nucleic acid extraction and RT-PCR

Total nucleic acid was extracted from CTV infected, leaves and stems of citrus plants (Zhou et al. 2001). For cDNA synthesis 100 ng of total RNA was denatured at 95 °C for 5 min in the presence of buffer, dNTPs and reverse primer followed by chilling on ice. RT reaction was carried out for 30 min at 42 °C in 20 μL of reaction mixture. For PCR amplification 1 μL of cDNA was used as template in reaction containing Taq DNA polymerase (Takara Bio, Otsu, Shiga, Japan), (1.25 U/50 μL) and gene p25 specific primers (forward: 5′ ATG GAC GACGAA ACA AAG 3′), (reverse: 5′ TCA ACG TGT GTT GAA TTT 3′).

### SSCP analysis

Coat protein p25 gene fragments of CTV were used for SSCP analysis. 9 µL of denaturing solution (20 mM EDTA pH 8.0, 95% v/v deionized Formamide, 0.25% w/v Bromophenol blue and 0.25% Xylene-cyanol), was mixed with 1 µL of 10 times diluted PCR product and heated for 10 min at 100 °C followed by chilling on ice. Samples (2.5–25 ng per well) were loaded on 12% non-denaturing polyacrylamide mini gel (10 × 8 cm) for electrophoresis using TBE buffer at 200 V for 3 h 30 min. During electrophoresis temperature was kept constant through water circulation. Silver nitrate solution was used to stain the gel followed by washing with ultrapure water twice, each time for 1 min, followed by washing with fresh sodium sulphide (0.2 mol/L) twice for 30 s. Sodium sulphide was changed and action sustained until bands were clear.

## Results

### Conformation thorough DTBIA

All 12 isolates were inoculated on six indicators plants collected from different citrus growing areas or districts of Punjab province of Pakistan and confirmed by DTBIA to know CTV infection. All 12 samples were found infected with CTV by DTBIA suggesting that CTV isolates could be inoculated successfully on all indicators plant (Table 1). Jatti Khatti is widely used rootstock in citrus groves of Punjab, Pakistan.

### Biological indexing

**Fig. 1:**
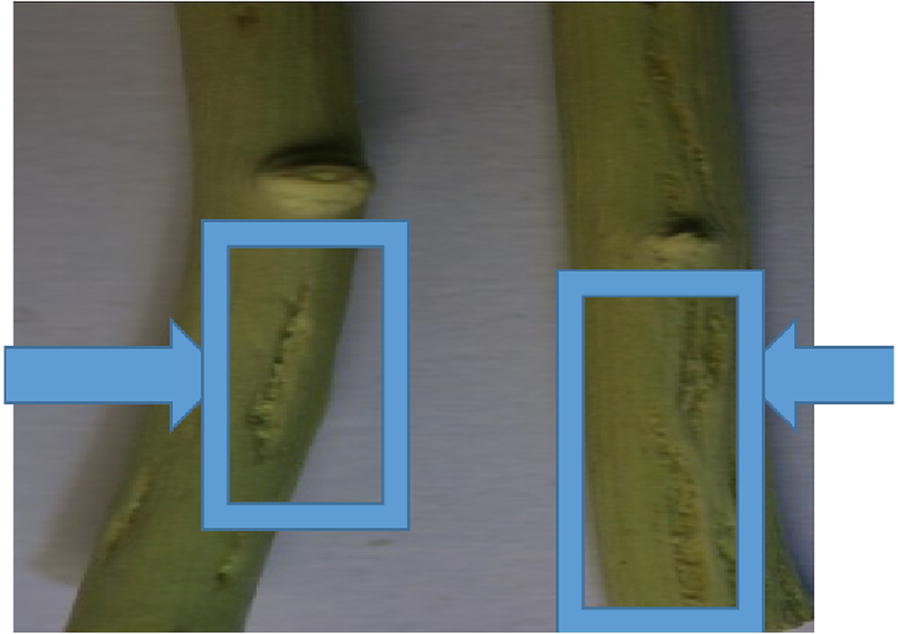
CTV isolate 21c causing sever stem-pitting on Mexico lime grafted on Fairchild

After 12–18 months of graft inoculation, biological indexing results were analyzed and symptoms expression was scored with reference to standardized scale (Garnsey et al. 1987b). Results of most of isolates depicted by biological indexing showed sever symptoms expression on Mexican lime, ranging from moderate to severe stem-pitting, while some isolates like 1AF, 21c and 5MF induced mild or moderate stem-pitting symptoms on different indicators, also developed severe reaction on Mexican lime (Fig. 1).

**Fig. 2:**
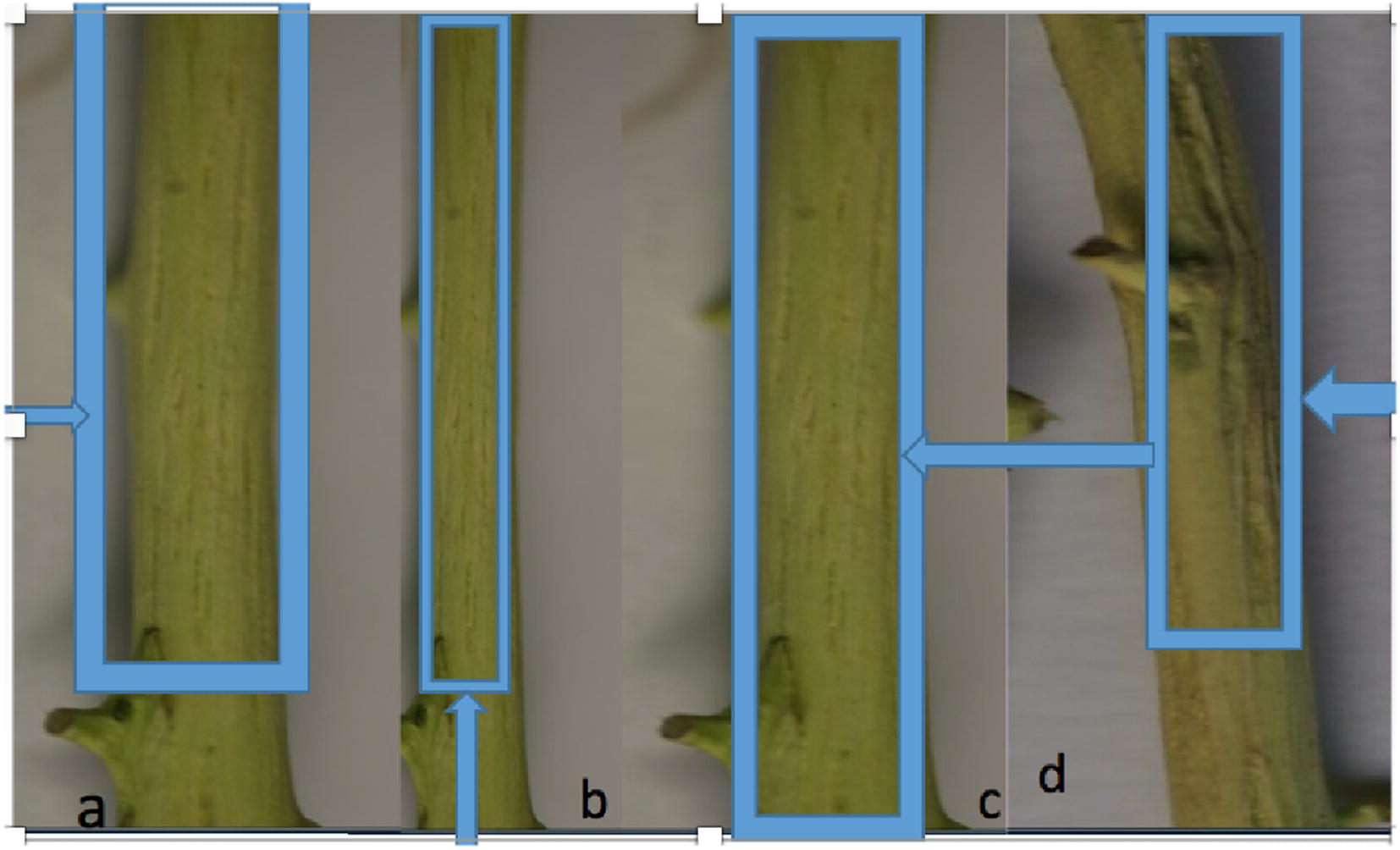
**a**, **b** CTV isolate 1AF and 5MF causing severe stem-pitting symptoms on Mexico lime; **c**, **d** CTV isolate 10GS and 21c causing severe stem-pitting symptoms on Duncan grapefruit grafted on sour orange

Symon sweet orange grafted on sour orange produced moderate stem-pitting symptoms on two of the CTV isolates 1AF and 5MF, while other isolates showed no symptoms. Duncan grapefruit grafted on sour orange showed severe stem-pitting on three [1AF, 21c and 11GS (Fig. 2)] of the isolates tested while moderate stem-pitting appeared on two isolates i.e., 5MF and 9KBS whereas other isolates are mild isolates that can be used as potential MSCP in future. Fenghuang pummelo grafted on pummelo also did not show any sever symptoms, only two isolates 1AF and 2KF showed mild symptoms. Some of the fenghuang pummelo plants died because of the higher dose of insecticide or damage caused by slugs. Mild to moderate response was found on Sour orange seedlings. Seedling yellows was seen in only two of the isolates (1 and 2) and uninoculated control plants remained healthier than all inoculated plants. Results of Symon sweet orange seedling and Duncan grapefruit remained more or less similar. Two isolates showed moderate while one isolate showed severe stem-pitting in the plants. Three CTV isolates did not produce any symptoms on biological indicator plants whereas showed positive result with DTBIA, indicating that these CTV isolates are mild strains. While three CTV isolates i.e., 4MF, 8KBS and 10GS showed mild symptoms only in Mexican lime suggesting that these isolates could be use effectively for cross protection. (Table 2).

**Table 2:** Response of indicator hosts graft inoculated by CTV isolates

CTV isolates	Indicators					
	Fenghuang pummelo on pummelo	Duncan grapefruit on sour orange	Sour orange seedlings	Mexican lime on tangerine Fairchild	Symons sweet orange on sour orange	Symons sweet orange seedlings
1AF	+	SY	+	+++	++	++
2KF	+	+	+	++	_	+
3MF	_	+	+	++	_	+
21c	_	+++	++	+++	_	+++
4MF	_	_	_	+	_	_
5MF	_	++	+	++	++	+
6MF	_	+	SY	++	_	++
7KBS	_	+	+	++	_	+
8KBS	_	_	_	+	_	_
9KBS	_	++	++	++	_	++
10GS	_	_	_	+	_	_
11GS	_	+++	+	++	_	+

+: mild, ++: moderate, +++: sever, SY: seedling yellows

**Fig. 3:**
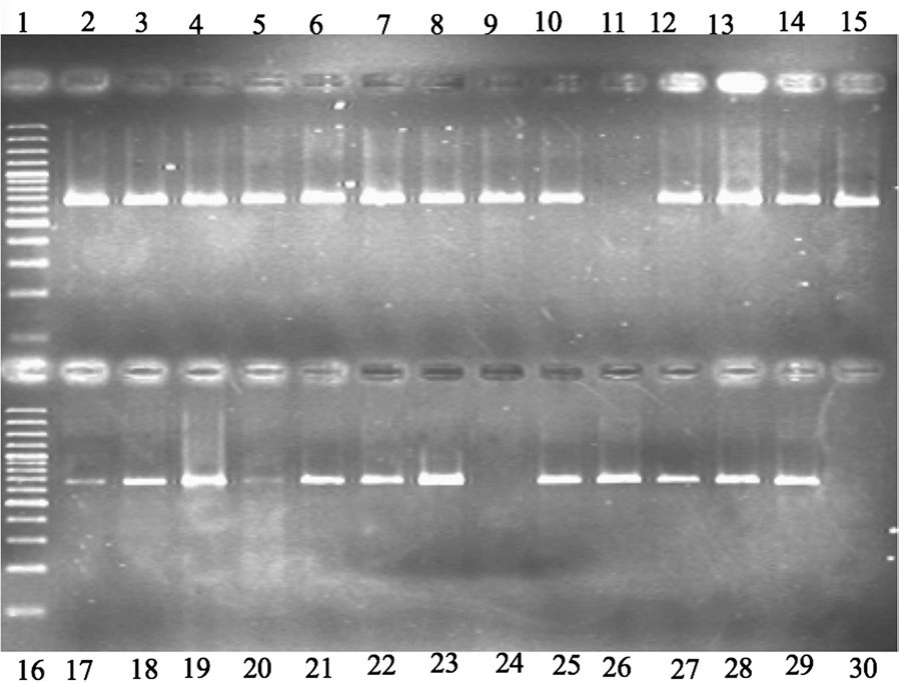
RT-PCR products of CP gene about 672 bp. In lane 1 and 16 DNA marker with 100 bp ladder; while lane 11 and 24 CTV-negative sample (by DTBIA); 29, positive control; 30, negative control and rest of the samples from 8 citrus growing areas of Punjab were also positive by DTBIA

**Fig. 4:**
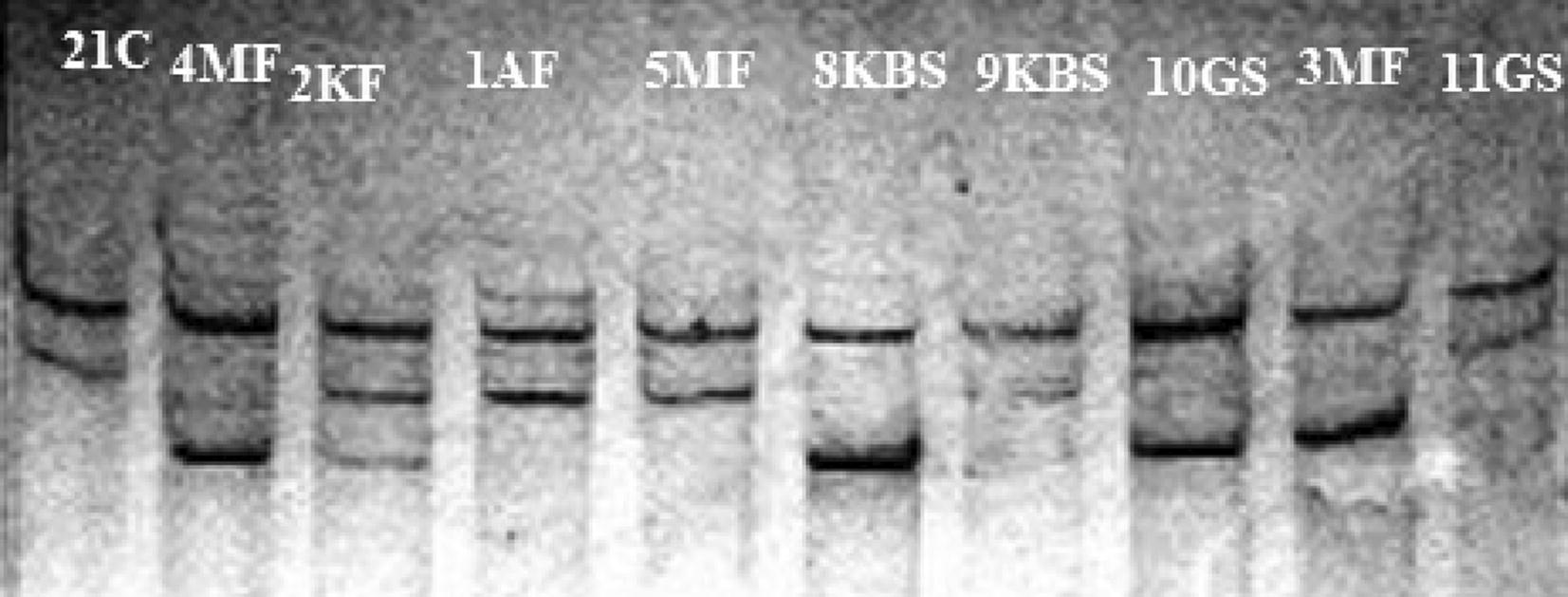
Gel showing results of SSCP analysis of the p25 gene of CTV isolates used in this study. Close bands correspond to severe strain whereas apart bands represent mild strain of CTV

All the grafted indicator plants were also tested by RT-PCR for confirmation (Fig. 3). Some isolate which showed no response on indicator plants like Fenghaung pummel grafted on pummelo and Symon sweet orange grafted on sour orange showed positive results when tested with RT-PCR. They will be considered as mild strain isolates. CTV mild and sever strains were further identified characterized and confirmed by p25 gene analysis from isolates using single-strand conformation polymorphism (SSCP) assay (Fig. 4). SSCP analysis represented that most of isolates were confined to only one predominant sequence variant. SSCP profiles of PCR amplified products from CTV isolates showed bands patterns corresponding to mild and sever strain. Distinct virus isolates could be separated either for cloning the coat protein genes or variants (haplotypes).

### Discussion

Different CTV strains induce variable symptoms on various citrus cultivars and rootstocks on which citrus scion is grafted and propagated. Prominent symptoms are not observed on most of CTV strains on commercial citrus cultivars because of their mild nature. Symptoms can only be detected by biological indexing of indicator hosts plants like Mexican lime or through serological and nucleic acid tests. Seedling yellows (SY) is severe strain of sour orange, grapefruit and lemon but when these seedlings are grown under controlled conditions, this SY strain causes dwarfing and severe chlorosis. When citrus cultivars such as grapefruit, sweet orange and other grafted on sour orange rootstock, leads to quick decline of trees within few weeks upon inoculation with severe strain of CTV. Declined trees always show yellowing or browning of leaves (Garnsey et al. 1987a; Brlansky et al. 2002; Dolja et al. 1994). Decline inducing CTV strains cause phloem necrosis at graft union on citrus trees propagated on sour orange rootstock. Beside these mild and decline-causing CTV strains, other severe strains cause stem-pitting (Ghorbel et al. 2000).

In this study, 12 CTV isolates were collected from Punjab province of Pakistan and characterized by biological indexing. We maintained all the isolates in green house under controlled day and night temperatures; high voltage electric bulbs were used to overcome the effect of long cloudy days. Our findings showed that mild to severe strains of CTV were present in field. We found that severe stem-pitting strain of CTV is predominant which correlates to our previous findings that 50% of sweet orange trees in Pakistan are infected with CTV and show stem-pitting symptoms (Atta et al. 2012, 2017). Bio-characterization of CA-VT-AT39 (Dekopon-AT), P108A (Dekopon), and VT-RH isolates induced severe symptoms of seedling yellows and stem-pitting (Yokomi et al. 2018) while another study showed that CTV strains rarely exhibited seedling yellows (SY) in grapefruit and sour orange seedlings (Yan et al. 2007), this may be explained as SY inducing CTV strains were progressed through seedling yellows sensitive hosts, like sour orange and grapefruit. (Yang et al. 1999). Our study depicted rare SY in all Duncan grapefruit sour orange, and fenghuang pummelo. Mexican lime was only indicator showing severe stem-pitting and vein clearing by maximum grafted isolates. Mild to severe stem-pitting appeared only in few plants such as Symons sweet orange and Duncan grapefruit. Except in Mexico lime most of the isolates did not show conspicuous symptoms in any of the indictor’s cultivars suggesting that most of isolates from Pakistan are mild and mild to moderate, only one or two isolates were found to be severe. SSCP assay confirmed the occurrence of inconsistency between and mild isolates under study, whereas isolates were mild to severe. Whereas, CTV isolates from Egypt did not differ significantly and each isolate consisted of very similar haplotypes (Amin et al. 2006). Effectiveness of MSCP to decrease the effect of severe strains has been reported by many scientists. Now we have enough information regarding different strains of CTV prevailing in Pakistan. Best to our understanding, most of the CTV isolates from Pakistan are mild isolates and are the good base to be used in MSCP in Pakistan and there is a need for proper indexing of mother trees and a virus-free propagation scheme in Pakistan in near future. Before the sever strain of CTV become epidemic, we have to deliver in field the outcome of this study. Further this study would help in citrus certification program in Pakistan by developing citrus plant tolerant to severe strains of CTV through introduction of MSCP.

## Data Availability

The authors declare that all data supporting the findings of this study are available from the corresponding authors upon request. CTV isolates can be requested from Prof. Dr. Changyong Zhou.
